# Physical Education in America

**Published:** 1890-11-01

**Authors:** 


### Physical Education in America.

We are rather surprised to find that so little is done in American schools in the way of systematic physical training. In colleges for young men, indeed, athletics are much thought of, the students at Harvard College entering into them with such enthusiasm and energy, that the college faculty found it advisable, two or three years ago, to give attentive study to the matter. The exhaustive report which was the outcome of their inquiries admits that "there are many excesses and evils connected with athletic sports, as intensified by inter-collegiate competition," and suggests certain alterations; but the President adds that "the faculty take comfort in the general physical improvement of the average student, and hold that dyspepsia is less tolerable than a stiffened knee or thumb, and that effeminacy and luxury are even worse evils than brutality." Which reminds us of the answer given by the headmaster of one of our English public schools to an anxious mother, who asked whether he did not consider playing football, under the present rules, a very bad thing :—"My dear madam, I know of but one thing worse." "And that is——" "Not playing football."

At Amherst College the department of hygiene and physical education is under the charge of two physicians, who minutely examine each student when he enters the college, and twice again during his collegiate course, and advise him as to the particular course of training which he should take. Statistics as to the health and strength of the students have been kept for over twenty years; and it is interesting to learn that, while the health of a young man from fifteen to twenty-five years of age is apt to decline, the Amherst students show a marked increase in health during the successive years of their course. The faculty of Harvard College have now informed holders of scholarships that they are expected to present themselves twice in the year before the director of the gymnasium, to be examined as to their physical condition, and to receive suggestions with regard to it. Surely it would be well if this rule were extended, so as to embrace all the students. To overdo physical training is as bad as to neglect it; and without careful supervision there will always be boys, both in America and in England, of slender physical powers, who, in their great anxiety to do all that their comrades do, will injure their health, and perhaps even endanger their lives.

American girls, on the other hand, appear to have but little provision made for them in the way of physical education. In only one of the colleges open to men and women alike, do we hear that gymnasium classes are held for the lady students; and of all the two hundred and seven colleges and seminaries exclusively for women, the Abbot Academy at Andover is the only one reported by the Commissioner of Education as having a gymnasium. Harvard Annex itself, in spite of its close connection with Harvard College, has, as yet, no provision for physical training, though it has lately secured enough land for several additional buildings, one of which is to be, we are told, a gymnasium.

As to high schools, military drill and calisthenic exercises are given in a considerable number of them, and some are provided with proper gymnastic apparatus, though the number of these is limited, and increases but slowly. Elementary pupils are less well cared for. No city provides systematic physical training in its elementary schools; and manual training, though excellent in its way, by no means supplies the want, more especially as regards the girls. We cannot be surprised to hear that complaints are constantly being made by superintendents and teachers of the lassitude and want of energy of both high school and elementary pupils, and that a considerable proportion are "unnaturally small, delicate, and childish in appearance," their forms bent and feeble, and losing every day in muscular vitality.

It is, however, evident that the American nation is waking up to the realisation of the importance of this matter, and that the next few years will see a marked difference in the place accorded to physical education. Such excellent results have been produced by the unassisted efforts of certain high school pupils to secure for themselves gymnastic exercise, that the authorities have been led to consider whether it might not be well to give their official support; and in several schools gymnasiums have lately been opened, provision being made for girls as well as boys. The President of the Board of Education for Yonkers, N.Y., pronounces that a thorough system of physical development is greatly wanted, and mentions that a small sum for the purchase of apparatus has been included in the estimates for the coming year, while a Columbian superintendent urges that special teachers of health-exercises should be employed, their periodical visits to the schools being supplemented by instructions given by the regular teachers.

"The first step," says a New Jersey superintendent, "is to provide competent instructors to teach and train the pupils of the normal schools, and the teachers in the schools." And, turning to the Commissioner's report on the training of teachers, we find that physical education in normal schools is strongly advocated, not only for the sake of the pupils hereafter to be taught by students at these schools, but for the benefit of the students themselves, both now and with regard to their after life. Several school superintendents speak very strongly on this question. "The end for which this instruction is given," says one, "is that the graduates may have sound bodies, and so be able to teach a school better. No teacher with a weak body and resulting infirmities of mind can long retain the confidence of children." And another superintendent pronounces that "good physical strength and ready flow of animal spirits are requisites in the teacher, that he may be able to withstand the nervous strain, and command the attention and respect of his pupils." We are glad to see that gymnasiums are not uncommon in these normal schools, and that calisthenic exercises are generally made a part of the school work.

We cannot close this article without a brief reference to "Arbor Day," which has become quite an institution in many of the States during the last few years. On a certain day in the spring, appointed by the Governor himself, each school sallies forth, very often accompanied by a large number of the townspeople, and spends the whole day either in laying out and beautifying the school-grounds, or in planting forest, fruit, or shade trees, according to the need for them, in the neighbourhood. We think the idea an excellent one, for the children are encouraged to do everything with their own hands, and thus is inculcated a love for outdoor labour, and a taste for beautiful surroundings, which may be expected to last through life. Moreover, the actual work done on Arbor Day is very useful, and by no means contemptible in amount. In States such as Maryland, the beautifying of the neighbourhood is all that need be thought of, the children planting silver maples, tulip poplars, etc., in the parks; but in the "treeless regions of the west," where many forests have been recklessly destroyed, forest culture is now seen to be of great importance, and is warmly taken up by the schools, hundreds of groves having sprung up in Nevada, Nebraska, and other States since the institution of Arbor Day.

Could the delightful schoolboy-prig in *My Lady Nicotine* (delightful we mean, in a book—pray, Mr. Barrie, do not take this to mean that we are longing for an introduction !) have lighted upon an account of an American Arbor Day, before writing to his uncle that—"We walks in Hyde Park, admiring the works of nature, and keeps up our classics when we see a tree by calling it 'arbor,' and then going through the declensions; but we never climbs trees for fear of messing the clothes bestowed upon us by our beloved parents in the sweat of their brow, and we scorns to fling stones at the beautiful warblers which fill the atmosfere with music." Who shall say?

